# How to control the economic burden of treating cardio-cerebrovascular diseases in China? Assessment based on System of Health Accounts 2011

**DOI:** 10.7189/jogh.10.010802

**Published:** 2020-06

**Authors:** Yalan Zhu, Chunping Liu, Luwen Zhang, Quan Fang, Shuang Zang, Xin Wang

**Affiliations:** 1 College of Humanities and Social Sciences, China Medical University, Shenyang, China; 2 Administration School, Hainan Medical University, Haikou, China; 3 School of Health Services Management, Southern Medical University, Guangzhou, China; 4 School of Nursing, China Medical University, Shenyang, China

## Abstract

**Background:**

In the past two decades, chronic non-communicable diseases have become the leading disease burden, and cardio-cerebrovascular diseases (CCVD) are the main causes of death in chronic diseases. It has become the focus of global public health attention, in this study, System of Health Accounts 2011 (SHA 2011) is used to calculate health expenditure, discuss its economic burden, and put forward countermeasures.

**Methods:**

Data were collected by multi-stage stratified random sampling and the medical expenses of patients with CCVD were calculated based on SHA 2011, from the dimensions of the financing plan, institutional flow, and service function. Correlation and regression analysis were conducted by controlling factors influencing hospitalization expenses. All analysis were conducted by software SPSS.

**Results:**

The current health expenditure (CHE) of CCVD in Dalian was 3.986 billion Yuan, accounting for 12.88% of the CHE (30.947 billion Yuan). The current curative expenditure (CCE) of CCVD was 2.947 billion Yuan. 40.39% of CCVD financing came from social medical insurance, and the proportion of family health financing was higher (39.06%). The expenditures were consumed by general hospitals and elderly patients.

**Conclusions:**

The expenditure burden of CCVD in Dalian was massive, and wasted the health resource severely. It is necessary for the government to adjust the financing structure, reallocate the expenses of CCVD, and make institutional flow and functional distribution more reasonable.

Cardio-cerebrovascular diseases (CCVD) refer to the ischemic and hemorrhagic diseases of the heart, brain, and body, caused by hyperlipidemia, blood viscosity, atherosclerosis, hypertension, among other factors. CCVD have high incidence, high disability rate, high mortality rate, high recurrence rate, and many complications [1]. CCVD have become the first leading cause of non-communicable diseases (NCD) deaths worldwide at over 50% [2]. At the same time, the ongoing global aging and disease spectrum transformation leads to the ever-growing NCD burden in low-and middle-income countries in the coming decades. Indeed, from 2007 to 2017, the number of deaths caused by CCVD increased by 21.1% globally [3] and the mortality caused by CCVD (17.8 million) ranked first in the world in 2017 [4]. CCVD have also caused onerous burdens to the health care systems in both developed and developing countries [5]. In general, the prevalence and mortality of cardiovascular diseases in China are still on the rise. In 2016, the mortality rate of cardiovascular diseases in rural areas was 309.33 per 100 000, and the mortality rate of cerebrovascular diseases was 158.15 per 100 000. The mortality of urban cardiovascular disease was 265.11 per 100 000, and the death of cerebrovascular disease was 126.41 per 100 000 [6]. CCVD are also the major chronic diseases that seriously threaten the health of Dalian residents and impose substantial financial burdens.

Studies on the health expenditure and financial burden caused by CCVD have been carried out worldwide. In Russia, cardiovascular costs (RUR1462 billion) accounted for 0.19% of GDP in 2009 [7]. In America, the estimated total cost associated with cardiovascular disease reached US$ 315.4 billion in 2010 [8], accounted for 2.10% of GDP. In Australia, direct medical costs for cardiovascular disease in 2011 amounted to Australian$ 11.5 billion [9], accounted for 0.58% of GDP. In Turkey, the economic burden of cardiovascular disease (US$ 10.2 billion) accounted for 1.18% of GDP in 2016 [10]. In China, the total hospitalization expenses for CCVD in 2016 was huge: acute myocardial infarction spent 19.0 billion Yuan, intracranial hemorrhage 25.4 billion, cerebral infarction 60.1 billion, with the average annual growth rate since 2004 was respectively 29.15%, 16.88% and 22.24% [11]. As to the proportion of CCVD expenditure to GDP, the economic burdens of residents are also high, and China is no exception. However, few studies have assessed health expenditure on CCVD in China. Most of the articles studied the CCE of a specific cardiovascular disease or cerebrovascular disease, but rarely discussed and analyzed the total expense burden of CCVD in a region.

Dalian is one of the most developed cities in Northeast China but with a high aging rate than nation [12]. The study the burden of CCVD expenditure and economic burden in Dalian is a prediction of the national aging development. In our study, we collected the inpatient and outpatient expenses of 326644 patients with CCVD in Dalian, and adopted the “System of Health Accounts 2011” (SHA 2011) method to further calculate the data. SHA 2011 [13] is a new medical and health accounting system, which retains the original triaxial relationship and designs three analysis interfaces to make up for the existing shortcomings and make the cost analysis among different regions comparable. In SHA 2011, current health expenditure is used instead of the original medical spending. Mobile medical treatment defray is different from medical treatment and health care defray, which does not include fixed assets defray. It is now considered the global standard for setting up national health accounts and has been adopted by most countries [14]. We first calculated the health expenditure on CCVD in Dalian from three dimensions: financing, institutional flow, service function; afterwards, we analyzed the influencing factors of health expenses. The burden of residents' health expenditure was first analyzed by financing situation, and reasonable health policies were put forward.

## METHODS

### Data sources

The data of this study were mainly from official sources, including *Dalian Health Financial Annual Report 2017*, *Dalian Health Statistical Yearbook 2017*, *China National Health Accounts Report 2017*, and *Dalian Health Accounts Report 2017*. Demographic data were obtained from *Dalian Statistical Yearbook 2017*. The yearbook and annual report were provided by the Health Commission of Dalian city. The data of patients’ medical expenses were collected from medical institutions in Dalian by sampling survey.

### Study sample

The outpatient and inpatient medical expenditures of health and medical facilities were selected as samples in Dalian in 2017. Multistage stratified cluster random sampling was designed by lottery-style drawings (prefecture-level cities), or computer programing (selection of streets, communities, and towns). The first stage was to choose sample areas from Dalian city based on taking full consideration into the perfection of health information management system (medical insurance system, patient diagnosis and treatment information recording system, medical and health expenditure details) and the level of economic development (GDP, national income, per capita national income). We select all regions except Changhai county (Its medical conditions were relatively backward, the medical information system was not perfect, so it was unable to obtain complete samples of patients' treatment information) as the sample areas (including the Zhongshan district, Xigang district, Sha Hekou district, Lv Shunkou district, Gan Jingzi district, Jinzhou district, Wa Fangdian district, Pu Landian district, Zhuanghe district) and Dalian municipal medical institutions, and public health institutions. In the second stage, one general hospital, maternal and child health care hospital, disease prevention and control center, and traditional Chinese medicine hospital were randomly selected from each region. A total of 5 community health service centers were selected from each district, 20 township health centers were selected from each county-level city, 17 clinics and outpatient departments were selected from each district, and 3 villages were selected from each township as samples. In the third stage, a total of 456 health and medical facilities and professional public health institutions were randomly sampled in the selected sample areas according to the categories of health institutions and administrative levels. A total of 370 institutions with valid data were collected, including 53 primary health centers and community service centers, 284 village clinics and clinics, 5 maternal and child health care centers, 4 traditional Chinese medicine hospitals, 4 specialized hospitals, and 20 general hospitals. Some of the remaining institutions had serious data deficiency and could not be filled in. The basic information for all outpatients and inpatients included age, gender, disease, type of medical institution, type of insured, season, expenditure, region, etc. The collected sample data were cleaned and screened according to the International Classification of diseases-10 (ICD-10) code of classification of diseases. The Vlookup function was used in excel to find and encode or delete the data that did not meet the requirements.

There were multiple diagnoses of the same patient in the survey data. In this study, only the patients with the first diagnosis of CCVD were selected, without considering other complications. A total of 326644 valid items for CCVD from January 1 to December 31, 2017 were collected. The data included the new CCVD patients in 2017 and the patients diagnosed before 2017 but still treated in 2017. Finally, this data was established in the normalized database.

### Quality control and data management

Data gathering was classified and coded according to ICD-10. Data extract, audits, cleaning, and calculation were maintained by implementing the basic accounting guidelines of SHA 2011, and a serious of uniform training. That is, the staff involved in the data cleaning procedures were trained by the National Health Commission of China and evaluated through an examination. All data were entered electronically into a data terminal that was directly connected with the Stata 12.0 (Stata Corp, College Station, TX, USA).

### Research methods

The accounting principles and methods adopted in the study were according to the revised edition of “A system of health accounts 2011” [15]. Stata 12.0 process was used to analyze the information of patients with CCVD.

### Curative expenditure accounted

Current curative expenditure includes medical income, government basic expenditure subsidy and government project subsidy income, which are divided into outpatient and inpatient parts. Taking outpatient expenses of CCVD as an example (there is no project subsidy for this disease, so the calculation is omitted), outpatient curative services (S_OCS_) includes outpatient curative income (S_OCI_) and outpatient basic curative expenditure subsidy (S_OBS_), and the calculation formula is as follows:

S*_OCS_* = S*_OCI_* + S*_OBS_*

Note: the outpatient income of preventive is excluded (the outpatient income in the hospital financial accounting includes the outpatient treatment income and the outpatient income of preventive service, so the outpatient income of preventive service needs to be excluded in the calculation).

Among them, S_OCI_ represents direct medical health expenditure includes treatment fees, medicine fees, diagnosis fees, nursing fees, bed fees, etc. S_OBS_ represents that, to ensure the normal operation of the institution and the completion of daily work tasks, the subsidy provided by the finance mainly includes personnel funds and public funds, and the security provision for curative services.

Outpatient curative income:


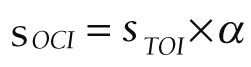
,


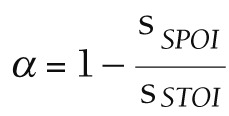
,

where S_TOI_ represents the total outpatient income, α represents the proportion of the outpatient income of sample institutions without preventive expenditure, S_SPOI_ represents the preventive outpatient income of sample institutions, and S_STOI_ represents the total outpatient income of sample institutions.

The outpatient curative cost of each patient is recorded as *a_i_*, the total cost is *a*, the sharing coefficient of each patient is *δ_i_*:


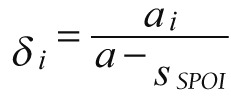
,

and the sharing coefficient is allocated to each case:


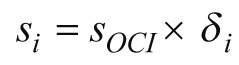
.

According to this method, the multi-dimensional distribution and composition of curative expenses are divided according to institution, age, gender and disease, and the expenses is:


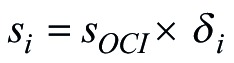
.

Outpatient basic curative expenditure subsidy:


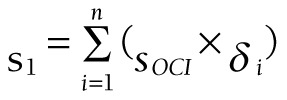
,


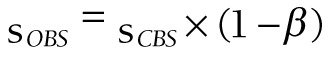
,


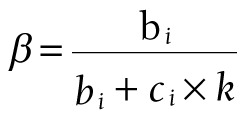
,

Among them, S_CBS_ represents the curative basic expenditure subsidy, *b_i_* represents the total number of inpatient bed days, *c_i_* represents the number of outpatient curative patients, k = 0.1 (representing 10 outpatient visits equivalent to 1 inpatient bed day), *d_i_* represents the total number of outpatient visits, *e_i_* represents the number of preventive outpatient patients of sample institutions, *f_i_* represents the total number of outpatient visits of sample institutions.

The subsidy is allocated to each case by coefficient *δ_i_*:


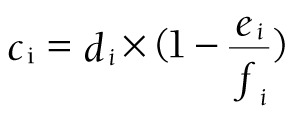
.

According to this method, the multi-dimensional distribution and composition of therapeutic basic expenditure subsidy are divided according to institution, age, gender and disease, and the expenses is:


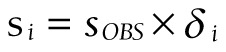
.

The outpatient income, outpatient visits, and basic expenditure subsidy are from the total sum of health statistics/financial annual report; the proportion of preventive outpatient income to total income and the proportion of preventive outpatient visits to total outpatient visits are from data of the sample institutions.

The calculation method of inpatient curative services is similar to that of outpatient services, but the slightly different is that the calculation of inpatient does not exclude preventive services. Finally, we can calculate the inpatient curative income and the inpatient basic curative expenditure subsidy as follows:


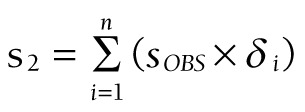
,


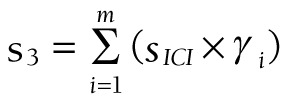
,

where *γ_i_* is the sharing coefficient of inpatients in the sample.

The final curative expenditure calculated by age, gender, disease, etc., is:

S*_CCE_* = S*_1_* + S*_2_* + S*_3_* + S*_4_*

### Other expenditure

The method to calculate prevention expenditure is based on the comparison between income and expense of each prevention project. If income < expense, take service expense to calculate, otherwise take service expense. Assistant services, medicine cost, management cost should also be brought into accounts, each item divided into government, social or family financing scheme.

### Analysis of influencing factors of CCVD expenditure

Because the inpatient expenditures do not follow the normal distribution, the regular distribution test was done for inpatient expenditures. Then the abnormal distribution test was used to analyze the data after logarithmic transformation. Data were typically distributed after logarithmic transformation. Multiple stepwise regression was used to analyze the influencing factors. The independent variables were age, gender, length of stay, hospital type, region, payment method, and operation. Inclusion criteria were 0.05 and exclusion criteria were 0.10.

All statistical analyses were performed using SPSS software (version 24.0) (IBM, Armonk, NY, USA).

## RESULTS

### General situation of expenditure

Current health expenditure was the monetary expression of residents' medical and health service consumption, excluding capital investment such as fixed assets construction. In 2017, CHE of CCVD in Dalian reached 3986.49 million Yuan (73.94%), 2947.62 million Yuan CCE (13.69%), 20.38% of chronic diseases (14.462 billion Yuan), and 0.40% of the GDP.

### Allocation of CHE in different service functions

In 2017, among the component of CHE of CCVD in Dalian, the expenditure of treatment services was the highest at 2947.62 million Yuan (73.94%), followed by the proportion of treatment expenditure in the CHE in all the diseases. The outpatient and inpatient expenditure were 757.98 million Yuan (19.01%) and 2189.64 million Yuan (54.93%), lower than the outpatient expenditure in CHE. The expenditure of medical supplies was 592.29 million Yuan, accounting for 14.86%, which was lower than the proportion of medical supplies in CHE. The expenditure of preventive services, health administration and financing management, accounted for 9.03% and 2.04%, respectively, both higher than the proportion of CHE (**Table 1**).

**Table1:** Composition of cardio-cerebrovascular diseases of current health expenditure (CHE)

Service function	CHE of cardio- cerebrovascular diseases (100 million Yuan)	Proportion (%)	CHE (100 million Yuan)	Proportion (%)
Treatment:	29.47	73.94	215.27	69.56
-Outpatient	7.58	19.01	77.83	25.15
-Inpatient	21.90	54.93	137.44	44.41
Medical supplies	5.92	14.86	59.63	19.26
Ancillary services	0.05	0.13	1.23	0.4
Preventive services	3.60	9.03	27.66	8.94
Governance, health administration and financing management	0.81	2.04	5.68	1.84

### Allocation of CCE in different medical institutions

**Figure 1:**
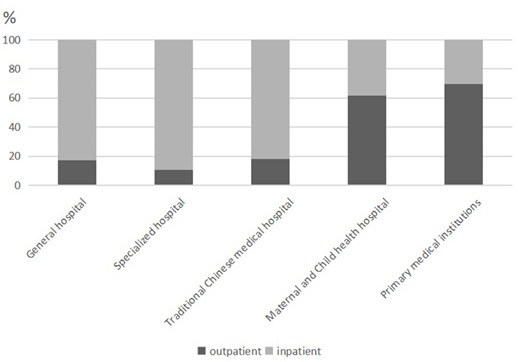
Allocation of outpatient and inpatient expenditures.

As to the distribution of expenditure, in 2017, 77.79% of the expenditure of treatment for CCVD were spent in general hospitals, 16.49% in primary hospitals, and very few in maternal and child health care hospitals and specialized hospitals. Among the CCE, the outpatient expenditure was 757.98 million Yuan (25.72%), and the inpatient expenditure was 2189.64 million Yuan (74.28%), of which the inpatient expenditure in general hospitals was much higher than that outpatient expenditure; and the outpatient expenditure in primary medical institutions was higher than that inpatient expenditure, most of them occurred in outpatient institutions (**Figure 1**).

### Allocation of CCE in different diseases

**Figure 2:**
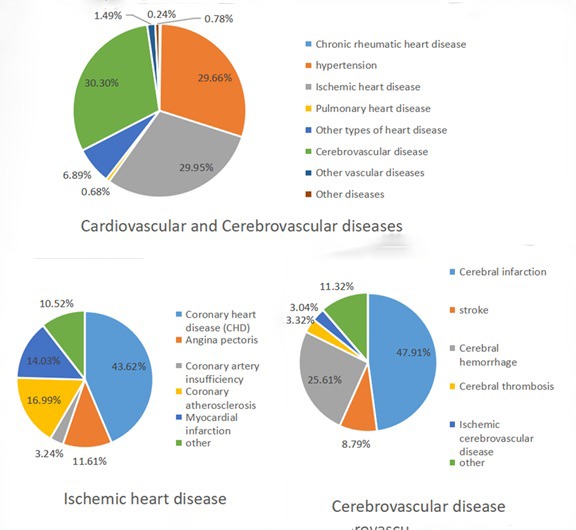
International Classification of Diseases (ICD) classification of current curative expenditure (CCE).

Among the diseases types, the CCE of hypertension, ischemic heart diseases, and cerebrovascular diseases accounted for 89.91%. Among all types of ischemic heart diseases, the expenditure of patients with coronary heart disease accounted for 43.62%, angina pectoris, coronary atherosclerosis. For cerebrovascular disease, the expenditure of cerebral infarction was at the dominant position, accounting for nearly 50%, followed by a cerebral hemorrhage, accounting for a quarter of the expenditure of cerebrovascular diseases (**Figure 2**).

### Allocation of CCE in different ages

**Figure 3:**
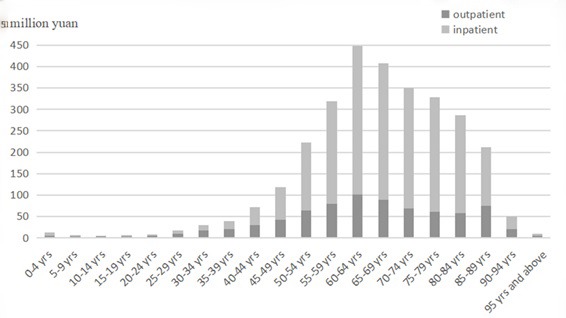
Age distribution of current curative expenditure (CCE).

From the age distribution of the CCE of CCVD, the CCE was mainly spent by population aged 50-89 years, accounting for 87.3%. However, the distribution of the expenditure of CCVD among people aged 20 and below and those aged 90 and above were relatively small. Second, the outpatient service proportion of patients with CCVD aged 5-39 years was higher than that of inpatient service, patients aged over 40 years old were mainly in inpatient service. Young patients were mostly treated in outpatient service, while middle-aged and elderly patients mainly were hospitalized (**Figure 3**).

### Health financing schemes

**Figure 4:**
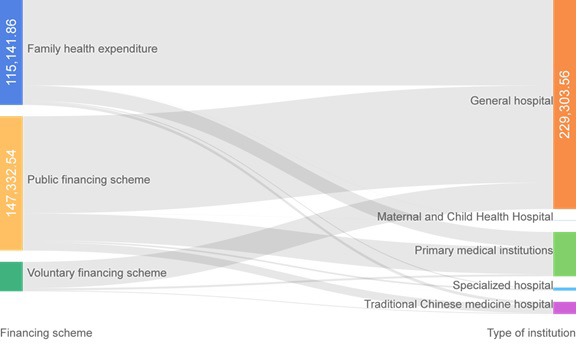
Flow of financing in different institutions.

From the perspective of financing structure, public financing accounted for 49.98% of the CCE of CCVD, among which government programs and social medical insurance accounted for 19.19% and 80.81% respectively. Second, family health expenditure accounted for 39.06%. From the different organization financing flow direction, the general hospital financing mainly depended on the individual and the public financing; hospitalization expenditure accounted for the majority of all funding (**Table 2**, **Figure 4**).

**Table 2:** Financing scheme for inpatient and outpatient

Service function	Public financing scheme (million Yuan)		Voluntary financing scheme (million Yuan)		Family health expenditure (million Yuan)
**Social medical insurance**	**Government financing**	**Commercial insurance**	**Enterprise financing**
Outpatient	171.49	116.01	30.02	24.3	416.16
Inpatient	1019.17	166.66	170.67	97.88	735.26

### Influencing factors to hospitalization expenditure

Multiple linear regression was used to analyze the influencing factors of hospitalization expenses in patients with CCVD. A standard test method was used to convert the total hospital expenses to a normal distribution, and there was a multicollinearity relationship between the variables. From the perspective of the standardized regression coefficient, the first three factors affecting hospitalization expenses were the length of stay, whether surgery was performed and whether there was insurance. Age, hospital level and gender also entered the regression equation. The linear model can explain the 61.4% change in total hospitalization expenses (**Table 3**).

**Table 3:** Regression analysis of influencing factors of hospitalization expenditure

	Unstandardization coefficient		Standardization coefficient	T	Sig
**B**	**SE**	**Beta**
Constant	-6380.878	892.879		-7.146	<0.001
Stayday	1224.077	11.192	0.526	109.374	<0.001
Surgery	4.326	0.088	0.246	49.014	<0.001
Age	-17.344	5.995	-0.038	-2.893	0.004
Insurance	374.696	41.818	0.043	8.960	<0.001
Gender	1164.691	145.747	0.038	7.991	<0.001
Institution level	534.202	215.493	0.033	2.479	0.013

*B = unstandardization regression coefficient. SE = Standard Error. Beta = standardization regression coefficient. *t* = t test value (t-statistic). Sig = significance (p) of coefficients.

According to Spearman correlation analysis, the correlation between the study variables was found, and the statistical analysis results were shown in **Table 4****.** The hospitalization expenses was positively correlated with the number of days of hospitalization (*r_s_* = 0.639, *P* < 0.001), surgical conditions (*r_s_* = 0.328, *P* < 0.001), gender (*r_s_* = 0.069, *P* < 0.001), and age (*r_s_* = 0.060, *P* < 0.001). Hospitalization expenses were negatively correlated with insurance and institution types (*r_s_* = -0.057, *P* < 0.001), (*r_s_* = -0.086, *P* < 0.001). Age was negatively correlated with gender and institutional level (*r_s_* = -024, -0.745, *P* < 0.001).

**Table 4:** Spearman correlation analysis among variables*

		1	2	3	4	5	6	7
**1**	Hospitalization expenses	1.000						
**2**	Stayday	0.639‡	1.000					
**3**	Surgery	0.328‡	-0.034‡	1.000				
**4**	Insurance	-0.057‡	-0.117‡	0.052‡	1.000			
**5**	Institution level	-0.086‡	0.014†	-0.355‡	-0.220‡	1.000		
**6**	Gender	0.069‡	0.020‡	0.024‡	-0.088‡	-0.002	1.000	
**7**	Age	0.060‡	0.009	0.261‡	0.196‡	-0.745‡	-0.024‡	1.000

*For surgery: no surgery = 0, and surgery = 1. For insurance status (sorted by reimbursement ratio): urban employees’ basic medical insurance = 1, urban residents’ basic medical insurance = 2, new rural cooperative medical care or urban and rural medical insurance = 3, self-funded = 4. For institution level: provincial hospital = 1, municipal hospital = 2, district and county hospital = 3, For gender: female = 1, male = 2.

†*P* < 0.05.

‡*P* < 0.01.

## DISCUSSION

CCVD are the leading cause of death and disability in the world. The World Health Assembly have called for the global efforts to reduce CCVD mortality by 25% before 2025 [16]. Reports about the growing prevalence of CCVD are worthy of attention. Health care and treatment consumed by increasing patients with cardiovascular disease have caused severe economic burdens. It has reported that total expenses for cardiovascular hospitalization have increased rapidly since 2004, with an inflation-adjusted annual rate of increase of more than 20%, much faster than the increase in GDP [17]. In 2017, the CHE in Dalian accounted for 4.2% of GDP, and the CCE of CCVD accounted for 0.40% of GDP, indicating that CCVD consume a large number of health resources. This is a severe phenomenon that cannot be ignored and the prevention and control system should be strengthened.

Age analysis revealed that the expenditure of CCVD was mainly distributed in by senior residents aged 50-89, especially among age group of 55-75, and the distribution of health expenditure is consistent with the prevalence of diseases [9]. Aging is an important factor affecting the incidence of CCVD and the rapid increase in expenditure [18]. In 2017, the elderly population (≥60 years old) in Dalian was over 24.99% of the total household registration population [19], much higher than the national level of 17.3% [20]. Elderly patients are constantly losing physical functions, and most of them suffer income loss. Once diseases attack them, even if the application is the most advanced and perfect treatment, still more than 50% of patients cannot provide for themselves completely [21]. For chronic elderly patients with CCVD should be given priority prevention and control.

As for the disease types, hypertension, cerebral infarction, coronary heart disease, cerebral hemorrhage, and coronary atherosclerosis are the top five key diseases, and the expenditure is the highest, so we should focus on the control of these diseases. At present, the spectrum of diseases has changed, and the health mode under the transformation has brought about the increase in residents' medical and health services utilization. From the perspective of overall utilization of health services, it is not only Dalian but also China, with circulatory system diseases in the first place and the top five chronic diseases are CCVD. This is similar to Yan Li’s study on chronic diseases of the elderly [22]. Therefore, it can be seen that CCVD have become the main diseases that endanger the causes and causes of death of residents and elderly people in Dalian and China. In our study, the medical expenditure of cerebral infarction, ischemic heart diseases, and hypertension were 2184.51 million Yuan (74.11%), and CCE was in direct proportion to the prevalence rate. Smoking, hypertension, unreasonable diet, and obesity are risk factors for CCVD [23]. Dalian municipal health planning in the future, therefore, need to emphasize the disease of blood vessels in chronic disease control strategy key health improvement projects and key projects for disease control and prevention, further perfecting the medical security system, improve the basic medical insurance compensation effectiveness, establish a link-to-link relationship between high-cost key diseases and health insurance. On this basis, CCVD intervention measures and effective control to reduce the incidence of disease burden. At present, the level of risk factors for CCVD in China is relatively high. This means that primary prevention activities need to be improved. Under the advocacy of healthy China 2030, the concept of health service should be changed from treatment centered to health centered. Promoting health education to boost national healthy quality; enforce early diagnosis and treatment to lower the morbidity risk of high-risk groups; reinforce standardized treatment to improve therapeutic effects [24]. Together with the world, China is committed to the prevention and control of chronic diseases, including CCVD, to promote the sustainable development of health undertakings.

As to the financing situation of health expenditure, public expenditure accounted for 49.98%, and the personal spending accounted for 39.06%, which is higher than the personal spending of the whole population with all diseases in Dalian (38.97%). During the 13th five-year plan, China set the goal of reducing the proportion of personal health expenditure to less than 30% of the total health expenditure. By 2020, personal health spending will fall to about 28% [25]. Now the individual economic burden of CCVD is much higher than this standard, and it is quite heavy. This is the same as the studies of Zanﬁna and Song [8,26]. The occurrence of CCVD will bring a heavy economic burden to individuals and families, but they can be prevented and controlled by taking active intervention measures. For example, we should completely implement the system of first diagnosis and blood pressure measurement for people over 35 years old, promote the co-management of “three highs”, carry out risk assessment and intervention guidance for high-risk groups such as overweight, obesity, high blood pressure, blood glucose, and dyslipidemia. It is also necessary to actively advocate a healthy lifestyle with low salt, low oil and low fat, regular physical exercise [27,28].

In the treatment of CCVD, hospitalization expenditure accounted for 74.28%, a review of the costs of disease research in 2009 found that major costs driver of direct cardiovascular disease costs was hospitalization (accounting for 61% of cost in Canada, 40%-45% in the USA and between 34% and 76% in Europe) [29]. Rapid growth in inpatient services is the main driver of increasing health spending [30], so it is meaningful to discuss the influencing factors. We found a significant relationship between the study variables and hospital costs, including the length of hospital stay, surgical insurance status, age, institutional level, and gender, which is consistent with other people's studies [31,32].

According to regression analysis, hospitalization days and patients' hospitalization expenses were positively correlated, and the most significant impact degree, which is in line with many other studies [33]. Hospitalization days not only reflect the severity of the diseases, but also reveal the comprehensive diagnosis and treatment of physicians is correct an indicator of the timely and effective, and can also indicate the hospital management level from the side [34]. To control health expenses, the hospital should improve the level of diagnosis and treatment levels and strengthen the management. The hospital should also widely adopt the clinical pathways, formulate an appropriate and orderly curative plans, shorten the time of preoperative hospitalization, and reduce unnecessary or repetitive diagnostic items [35].

The results of multivariate analysis showed that whether the surgical treatment in the hospital affected the expenses of hospitalization. Among the selected samples, 9983 (32.66%) patients underwent surgery, which account for 52.25% of the total expenses of treatment, and was significantly higher than that for non-surgical patients. Surgical treatment for patients with CCVD consume expensive stents and imported drugs. Therefore, relatively low-cost domestic equivalent products should be used instead of imported materials, and excessive use should be avoided as soon as possible. At present, surgical instrument catheters made by domestic manufacturers of high-value consumable medical equipment are entirely produced in accordance with international standards, and some or all of them can replace imported materials [36]. It is suggested that medical personnel try to use more domestic supplies, which is feasible in terms of economic and social benefits.

Medical insurance is a means of protection from the financial risk of incurring medical expenses. A medical insurance system is generally designed to improve accessibility and equity of health service utilization and to protect the citizen from medical impoverishment [37], which may also impact patient's expenses. If residents participate in medical insurance, their medical expenses are partially reimbursed by the insurance, so that the corresponding financial burden may be reduced. Medicare creates a link between the hospital and the patient. The expansion of health insurance will spur more people to get health care. Payments and co-payments related to the health insurance system will affect the number and manner in which hospitals and doctors provide services, as well as the amount, cost and process of care sought by patients with medical expenses [38]. In 2018, the coverage rate of social medical insurance in Liaoning province has been greatly improved, which is 94%. However, the reimbursement rate for chronic diseases is not high, suggesting that the level of residents' medical insurance compensation needs to be further improved. According to the results of domestic and foreign studies, it is difficult to reduce the proportion of household health expenditure solely by the increase in government health investment. In the case that the financial input of this region should be increased, the existing fund efficiency and financing level of social medical insurance should be improved to further reduce the proportion of household health expenditure. As the most economically developed city in northeast China, Dalian medical expenses growth quickly, it is necessary to improve the ability to raise funds to improve the social medical insurance in Dalian residents’ insurance system, encourage support residents to participate in commercial health insurance at the same time. The results of this study suggest that out-of-pocket payments account for a very high proportion of patients. In the future, the government should increase the ﬁnancial investment, raise reimbursement rates and set up differential reimbursements to meet the health needs of insured patients with different income levels [39].

### Limitations

First, the study only evaluated the economic burden of CCVD in terms of current curative expenditure. The economic burden of disease is a statistical index of economic expenditure and loss caused by all diseases and injuries in a region in a year. It includes direct medical expenses, indirect medical expenses, and other social losses. This article only discussed direct medical expenses, while some patients' indirect economic burden may be higher than that of direct medical expenses. Therefore, the economic burden we discuss is one-sided and cannot be regarded as the actual loss of patients. Second, only the patients with the first diagnosis of CCVD were selected, without considering other complications. In the process of treatment, doctors put forward treatment measures based on the comprehensive consideration of patients' conditions, so it is difficult to distinguish the costs of treatment, so it is difficult to separate the costs of other complications when calculating the economic burden of patients' disease.

## CONCLUSIONS

The use of SHA 2011 facilitated the interpretation of current health care spending patterns. CCVD patients in Dalian still face the problems of medical care coverage and access to medical services; their expenses are closely related to the length of stay, surgery, and insurance. This result indicates that it is of great significance to prevent the risk factors of CCVD and reduce the morbidity.
